# SarkoLife: quality of life in patients undergoing multimodal soft tissue sarcoma treatment

**DOI:** 10.1186/s12957-024-03632-x

**Published:** 2025-01-08

**Authors:** Sebastian Hoffmann, Tabea Hoffmann, Vlatko Potkrajcic, Christoph K. W. Deinzer, Katrin Benzler, Lars Zender, Adrien Daigeler, Johannes Tobias Thiel

**Affiliations:** 1 Department of Hand, Plastic, Reconstructive and Burn Surgery, BG Trauma Center Tuebingen, University of Tuebingen, Schnarrenbergstraße 95, Tuebingen, 72076 Germany; 2 Department of Marketing, Faculty of Economics and Business, University Groningen, Groningen, The Netherlands; 3 Department of Radiation Oncology, University Hospital Tübingen, Hoppe-Seyler-Str. 3, Tuebingen, 72076 Germany; 4 Department of Medical Oncology and Pneumology, University Hospital Tuebingen, Otfried-Mueller-Straße 10, Tuebingen, 72076 Germany; 5 University of Tübingen iFIT Cluster of Excellence (EXC2180) “Image-Guided and Functionally Instructed Tumor Therapies, Tübingen, Germany; 6 German Cancer Research Consortium (DKTK), Partner Site Tübingen, German Cancer Research Center (DKFZ), Heidelberg, Germany

**Keywords:** Sarcoma, Quality of life, SarkoLife, Sarcoma therapy, EORTC, Patient Reported Outcomes (PROs)

## Abstract

**Objective:**

To assess the tolerability of multimodal therapy in soft tissue sarcoma patients, particularly with regard to their quality of life and level of distress.

**Materials and methods:**

A retrospective cohort study enrolled individuals receiving sarcoma therapy at the sarcoma center of the University of Tuebingen between 2017 and 2022. Participants completed an online survey that included the EORTC’s questionnaire (QLQ-C30), coupled with the distress thermometer and demographic inquiries. The primary emphasis was on comparing three distinct modalities: Radiation, Chemotherapy and Surgery. The data were analysed performing one-way ANOVA.

**Results:**

A total of 237 patients were included in the study. There was a significant difference (*p* < 0.001) in quality of life according to the EORTC scores (high score = high quality of life) between the different treatments: chemotherapy (mean: 26.8 [standard deviation: 19.5]), radiotherapy (51.0 [21.5]), and surgery (46.9 [28.3]). Similarly, a statistically significant discrepancy (*p* < 0.001) was found in average distress levels (high score = high level of distress) corresponding to each treatment type: radiation (5.0 [2.7]), surgery (6.0 [2.9]), and chemotherapy (7.4 [2.4]). The rates of patients willing to undergo the same treatment varied across groups, with the highest percentage observed in the surgery group (94.2%), followed by radiation (87.4%), and chemotherapy (73.5%).

**Conclusion:**

Patients receiving multimodal therapy for soft tissue often find chemotherapy particularly demanding. Impairment of both quality of life and physical well-being is more likely and tends to be more severe compared with radiation or surgery. These observations should be taken into consideration when consenting patients and offering treatment plans.

## Introduction

Soft tissue sarcomas (STS) are a rare and highly heterogeneous group of tumors. These tumors can originate from nearly any type of soft tissue in the body, more than 80 different subtypes are known. These various subtypes are differentiated based on morphological, immunohistochemical, and molecular characteristics [1].

To confirm the diagnosis of a sarcoma entity, its grading and immunohistotyping a biopsy is required [2]. The tumor is typically graded using the FNCLCC system (Fédération Nationale des Centres de Lutte Contre le Cancer) [3]. Risk classification is based on grading, tumor size and depth to superficial fascia. Multimodal therapy should be offered to high-risk patients. This therapy includes components like radiotherapy, chemotherapy, and surgical procedures. Additional methods such as hyperthermia, angiogenesis inhibitors, or isolated limb perfusion (ILP) can be added as complementary measures [4, 5]. Despite all the further developments in recent years, R0 resection remains the most important therapeutic pillar [6].

The composition of the therapy and the individual treatment protocol for patients should be determined as specific individual decisions within the context of interdisciplinary tumor conferences. In addition to the psychological effects of being diagnosed with sarcoma, patients are also burdened by the side effects of the necessary, often initially curative therapy, which affects their quality of life [7]. Approximately one third of all cancer patients have comorbid psychological conditions, and for other malignancies, the subjective need for psychotherapy is reported to be around 40–50% [8, 9]. Several measurement instruments are available for the specific assessment of the quality of life of patients with malignancies. The EORTC Quality of Life Questionnaire is particularly well established. This instrument was developed in 1993 and has been revised several times since then [10, 11]. The current version, both psychological and somatic symptoms are assessed. Several scales (functional scale, symptom scale, and global health status) allow conclusions to be drawn about individual quality of life [12].

Despite existing studies on the topic of “Quality of Life in Sarcoma Patients,” the impact of this diagnosis on quality of life and psychosocial consequences is not clearly defined. Detailed studies that examine the patients physical and psychological well-being during therapy are needed [13].

The current research explores variations in the well-being of individuals with STS. Our hypothesis posits that there are disparities in the quality of life between patients undergoing three distinct treatments: surgery, radiotherapy and chemotherapy. Specifically, we anticipate, based on our clinical experience, that chemotherapy exerts the greatest influence on quality of life, followed by surgery and then radiotherapy. In clinical practice, it is common for patients to express concerns about the side effects of chemotherapy, with some individuals opting to discontinue treatment due to the associated discomfort. The aim of this study is to acquire in-depth knowledge regarding the quality of life throughout multimodal therapy for sarcoma, with the intention of informing collaborative decision-making with our patients concerning these specific treatment approaches.

## Methods

### Participants

All patients aged over 18 years with STS treated at the Comprehensive Cancer Center Tuebingen-Stuttgart (CCC) over a five-year period from 2017 to 2022 were included in the study. Given our primary focus on extremity sarcomas, tumors located in neck, thorax or abdomen or intracranially were not considered. The timeframe was chosen to allow for a mid-term retrospective analysis and to minimize selection bias, ensuring the inclusion of varied patient outcomes. Patients were contacted using the University of Tuebingen’s cancer registry via an information letter detailing the study, conditions and rights of participation, along with a link and QR code to an online questionnaire. A reminder letter was sent four weeks later. Participation required the ability to complete digital forms, as no paper questionnaires were available. Participants were interviewed retrospectively; the study did thus not affect the patient’s treatment. The patients were questioned about their recollection of personal well-being during each therapy modality (surgery, radiation, chemotherapy), without considering the duration elapsed since the therapy occurred. Notably some patients received more therapy modalities than others. Nevertheless, the data was sorted and analysed by modality in three groups: radiation, surgery and chemotherapy.

### Questionnaire

A web-based questionnaire (www.soscisurvey.de) was utilized for its compatibility with various browsers and mobile devices. A paper form of the questionnaire was not provided. The patients were recruited with an invitation letter delivered by mail. The opening page clarified the study’s objectives and voluntary participation. The initial section gathered demographic data, featuring mainly closed-ended questions with predefined responses. Some items, like age, allowed open-ended responses.

### Measures

The goal of the study was to investigate the quality of life after the treatments received at the CCC at the university of Tuebingen. We aimed to place a particular focus on the three different core modalities: radiation therapy, chemotherapy and surgery. We intend to assess the individual quality of life of patients at the time of each therapy. To answer this central question of the study, demographic data were collected in addition to using the validated EORTC QLQ-C30 and the distress thermometer. The EORTC scales range in score from 0 to 100, high scores representing high response levels. The distress thermometer utilizes a scoring system ranging from 0 to 10, with a score of 10 indicating the highest level of distress achievable. Moreover, patients were queried regarding their willingness to undergo the same therapy modality again (retake).

An a priori power analysis, conducted using G*Power software, determined that a sample size of between 100–200 subjects is required to achieve 90% power at a 0.05 significance level to detect the expected effect size in T-tests, F-tests, and Tukey–Kramer post hoc analyses.

As a comparison group, we selected tumor patients with various diagnoses in stages III-IV, as no cohort of patients with sarcomas was available. Values (standard deviation, median, mean) for the “Global Health Status” and “Physical functioning” scales were based on the “EORTC Reference Manual (from July 2008)” for the calculations.

### Statistical methods

Sample characteristics are presented for the total population in Table 1. Categorical variables are reported as numbers and percentages, and continuous variables as means with 95% CI.

**Table 1:** Baseline characteristics (specific items reported by patients)

	Frequency	Percent of total
	*n* = 237	
**Sex**		
Female	104	56.1
Male	133	43.9
**Age** (median)		
	61.0 (16.7)	
**Sarcoma Subtype**		
Dermatofibrosarcoma protuberans	20	8.4
Alveolar rhabdomyosarcoma	1	0.4
Alveolar soft tissue sarcoma	19	8.0
Angiosarcoma	5	2.1
Atypical lipomatous tumor	7	3.0
Biphasic synovial sarcoma	3	1.3
Chondrosarcoma	17	7.2
Clear cell sarcoma	2	0.8
Desmoid fibromatosis	4	1.7
Embryonal rhabdomyosarcoma	1	0.4
Epithelioid hemangioendothelioma	1	0.4
Epithelioid sarcoma	5	2.1
Fibrosarcoma	9	3.8
Giant cell tumor	2	0.8
Kaposi's sarcoma	6	2.5
Leiomyosarcoma	27	11.4
Malignant peripheral nerve sheath tumor	3	1.3
Mixed Tumour	1	0.4
Myxofibrosarcoma	10	4.2
Myxoid liposarcoma	11	4.6
Myxoinflammatory fibroblastic tumor	1	0.4
Pleomorphic dermal sarcoma	7	3.0
Pleomorphic liposarcoma	12	5.1
Sarcoma with BCOR gene alteration	1	0.4
Spindle cell sarcoma	16	6.8
Synovial sarcoma	5	2.1
Desmoplastic small round cell sarcoma	1	0.4
Extraskeletal myxoid chondrosarcoma	1	0.4
Extraskeletal osteosarcoma	1	0.4
High grade endometrial stromal sarcoma	1	0.4
Liposarcoma	21	8.9
Adenosarcoma	1	0.4
Myxoid Pleomorphic liposarcoma	1	0.4
CIC-rearranged sarcoma	1	0.4
Pleomorphic Rhabdomyosarcoma	2	0.8
Dedifferentiated liposarcoma	11	4.6
**Number of patients in each modality**		
patients with chemotherapy	67	23.2
patients with radiation	94	32.5
patients with surgery	225	94.9
**Frequency of modality per patient**		
1 modality	129	44.6
2 modalities	52.0	18.0
3 modalities	31.0	10.7
4 modalities	24.0	8.3
5 modalities	1.0	0.30

For all EORTC scales, participants’ scores were linearly transformed to a scale of 100 points according to the manual. A high score on the symptom scale indicates a high level of symptomatology. Distress scores were calculated according to the German manual [14]. A high score on the distress scale indicates a high level of distress. Withdrawal was measured using a binary yes/no choice.

Patient responses to the EORTC scales and distress scores were analysed using one-way ANOVA. In cases where Levene’s test revealed significant differences in variance, a Welch correction was applied. The effect sizes for the ANOVAs were quantified using eta-squared. Subsequent post hoc examinations were conducted applying Tukey corrections, and the effect sizes for these comparisons were calculated using Cohen’s d. Patient responses to the retake scale were analysed using a chi-squared test of independence.

The data were pre-processed using the dplyr package [15] (version 1.1.2; Wickham et al., 2023) in R (version 4.3.1; R Core Team, 2023 [16]) and analysed using JASP (0.17.3.0; JASP Team, 2024 [17]). Anonymized data sets and analyses scripts can be found at the online repository URL associated with this manuscript.

## Results

446 of the 1028 patients (43.39% response rate) invited to the study completed the questionnaire. Exclusions were made for 157 patients due to incomplete responses, 48 due to diagnostic discrepancies (including benign conditions or sarcoma location criteria not met), and 4 due to missing treatment information, leaving 237 patients (mean age = 52 [17]; 104 females, 133 males) eligible for analysis. Reasons for non-participation included 27 deceased patients, 111 undelivered invitation letters, and 497 unresponsive invitees.

Not all participants underwent all treatments, whereas some patients had a combination of therapies. In total, we counted 94 patients for radiotherapy, 67 patients for chemotherapy and 225 patients for surgery.

The average age of all 237 participants was 52 years (range 18–93 years). Among the participants, 133 were male, and 104 were female, indicating a balanced distribution (see Table 1).

77.6% of all patients described their current situation as tumorfree and under surveillance. 3.8% stated to be under curative therapy and 7.2% were undergoing palliative therapy. 11.4% did not know or could not determine their situation clearly.

There were 45 different subtypes (some very similar were summarized) of STS included in the study. The four most common subtypes were leiomyosarcoma, dermatofibrosarcoma protuberans, alveolar sarcoma, and liposarcoma (see Table 1). It is important to note that patients were asked about their diagnoses, but in general their statements were not cross-checked with clinical data.

### Frequency of modalities

Participants were asked about the number of therapy modalities they received. In addition to the three primary modalities of radiation, surgery, and chemotherapy, isolated limb perfusion (ILP) and hyperthermia were also considered. Among the participants, 108 received more than one therapy modality, while 129 underwent only one modality (Table 1).

### Distress, retake and quality of life

#### Distress

**Fig. 1:**
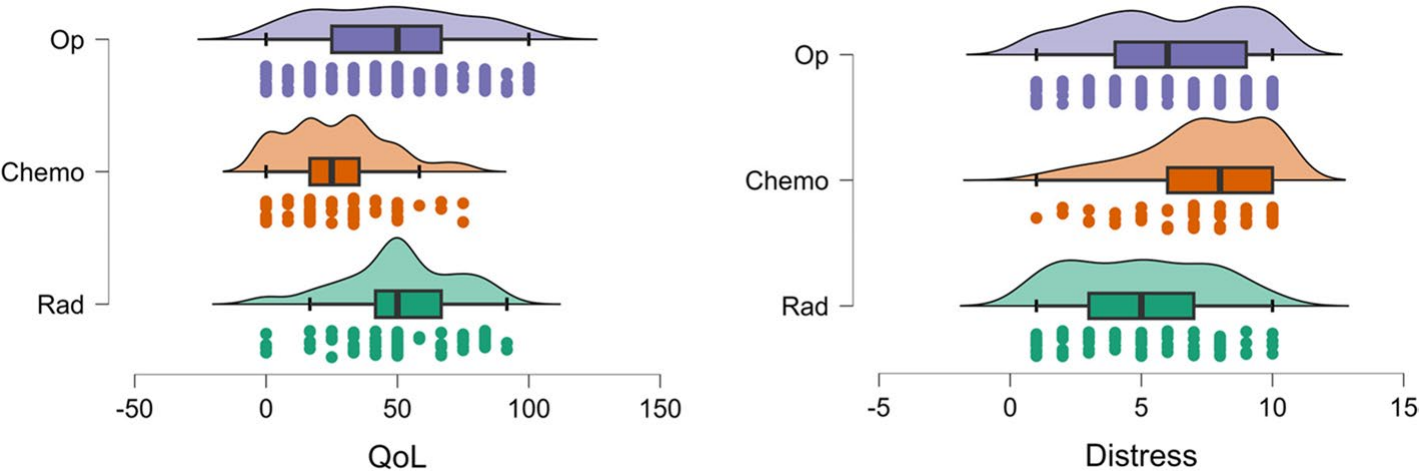
Mean distress scores for the three treatment types. Error bars visualise the 95% confidence interval

Figure 1 shows the mean difference in distress across different types of treatment. Participants reported the most distress for chemotherapy. We found a statistically significant difference in average distress according to treatment type (F (2, 169) = 18.306, *p* < 0.001, eta-squared = 0.07). A Tukey post-hoc test revealed significant pairwise differences between treatment types of radiation and chemotherapy with a mean difference of -2.408 (*p* < 0.001, d = -0.873), between radiation and surgery with a mean difference of -1.046 (*p* = 0.006, d = -0.379), and between chemotherapy and surgery with a mean difference of 1.362 (*p* < 0.001, d = 0.494).

#### Retake

A chi-squared test was conducted to examine the relationship between retake and treatment modality. The results indicated a significant association, χ^2^ (2, N = 427) = 26.209, *p* < 0.001. Retreatment rates varied by treatment modality, with the highest percentage reported in the surgery group (94.19%), followed by radiotherapy (87.38%), and chemotherapy (73.49%).

#### Quality of life

A statistically significant distinction was observed in terms of quality of life (F (2,183) = 31.96, *p* < 0.001, eta squared = 0.096). Subsequent Tukey post-hoc analysis revealed notable pairwise differences between treatment modalities. Specifically, there were significant differences between radiotherapy and chemotherapy, with a mean disparity of 24.1 (*p* < 0.001, d = 0.951), as well as between chemotherapy and surgery, with a mean difference of -20.05 (*p* < 0.001, d = -0.790). However, the discrepancy between radiotherapy and surgery, with a mean difference of 4.1, did not reach statistical significance (*p* = 0.389, d = 0.161).

#### EORTC

A comparison is made among radiotherapy, chemotherapy, and surgery in terms of global health, functional scales, and symptom scales. Table 2 illustrates the mean difference between the three modalities, together with the *p*-value from the Tukey post-hoc test and Cohen’s d. Table 3 presents a one-way analysis of variance, with group sizes of *n* = 95 for radiation, *n* = 68 for chemotherapy, and *n* = 225 for surgery.

**Table 2:** Oneway analysis of variants: comparison of the means of all three different conditions

	Radiation		Chemotherapy		Surgery		ANOVA
	*n* = 194		*n* = 67		*n* = 225		
	mean (standard deviation)						
**Distress**	5.0 (2.7)		7.4 (2.4)		6.0 (2.9)		*F*(2, 168) = 18.306, *p* < 0.001, eta-squared = 0.07
**Global Health**							
Quality of Life	51.0 (21.5)		26.8 (19.5)		46.9 (28.3)		*F*(2, 183) = 31.960, *p* < 0.001, eta-squared = 0.096
**Functional Scales**							
Physical Functioning	63.5 (25.6)		43.5 (25.4)		50.3 (32.8)		*F*(2, 176) = 13.490, *p* < 0.001, eta-squared = 0.050
Role Functioning	40.7 (34.1)		15.9 (26.8)		33.0 (35.0)		*F*(2, 172) = 14.843, *p* < 0.001, eta-squared = 0.055
Emotional Functioning	59.6 (27.4)		49.8 (29.9)		59.0 (31.6)		*F*(2, 385) = 2.715, *p* = 0.068, eta-squared = 0.014
Cognitive Functioning	74.4 (30.5)		54.7 (34.3)		73.1 (32.0)		*F*(2, 385) = 9.742, *p* < 0.001, eta-squared = 0.048
Socialc Functioning	54.2 (32.9)		36.8 (30.1)		55.4 (37.7)		*F*(2, 172) = 9.530, *p* < 0.001, eta-squared = 0.038
**Symptom Scales**							
Fatigue	53.0 (31.4)		76.8 (23.1)		52.5 (35.2)		*F*(2, 183) = 25.368, *p* < 0.001, eta-squared = 0.075
Nausea and Vomitting	12.5 (21.2)		41.2 (36.5)		10.4 (21.8)		*F*(2, 385) = 41.473, *p* < 0.001, eta-squared = 0.177
Pain	31.8 (32.5)		40.9 (32.6)		50.1 (35.9)		*F*(2, 385) = 9.703, *p* < 0.001, eta-squared = 0.048
Dyspnoe	24.2 (27.7)		47.5 (37.9)		27.7 (34.6)		*F*(2, 385) = 11.138, *p* < 0.001, eta-squared = 0.055
Insomnia	33.0 (33.2)		51.5 (34.8)		39.7 (37.6)		*F*(2, 164) = 5.813, *p =* 0.004, eta-squared = 0.027
Appetite Loss	24.2 (33.5)		53.9 (38.2)		20.4 (30.2)		*F*(2, 145) = 21.936, *p* < 0.001, eta-squared = 0.128
Constipation	14.7 (27.8)		37.3 (39.3)		16.7 (28.7)		*F*(2, 146) = 8.985, *p* < 0.001, eta-squared = 0.065
Diarrhea	11.9 (22.2)		25.5 (32.6)		9.8 (23.4)		*F*(2, 146) = 6.787, *p =* 0.002, eta-squared = 0.051
Financal Difficulties	24.6 (35.5)		33.3 (37.3)		22.4 (33.6)		*F*(2, 151) = 2.346, *p =* 0.099, eta-squared = 0.013
**Retreat**							
yes	90	87%	21	73%	227	94%	Chi-Squared = X^2 26.209, df 2, *p* < .001
no	13	13%	22	27%	14	6%	Chi-Squared = X^2 26.209, df 2, *p* < .001

**Table 3:**
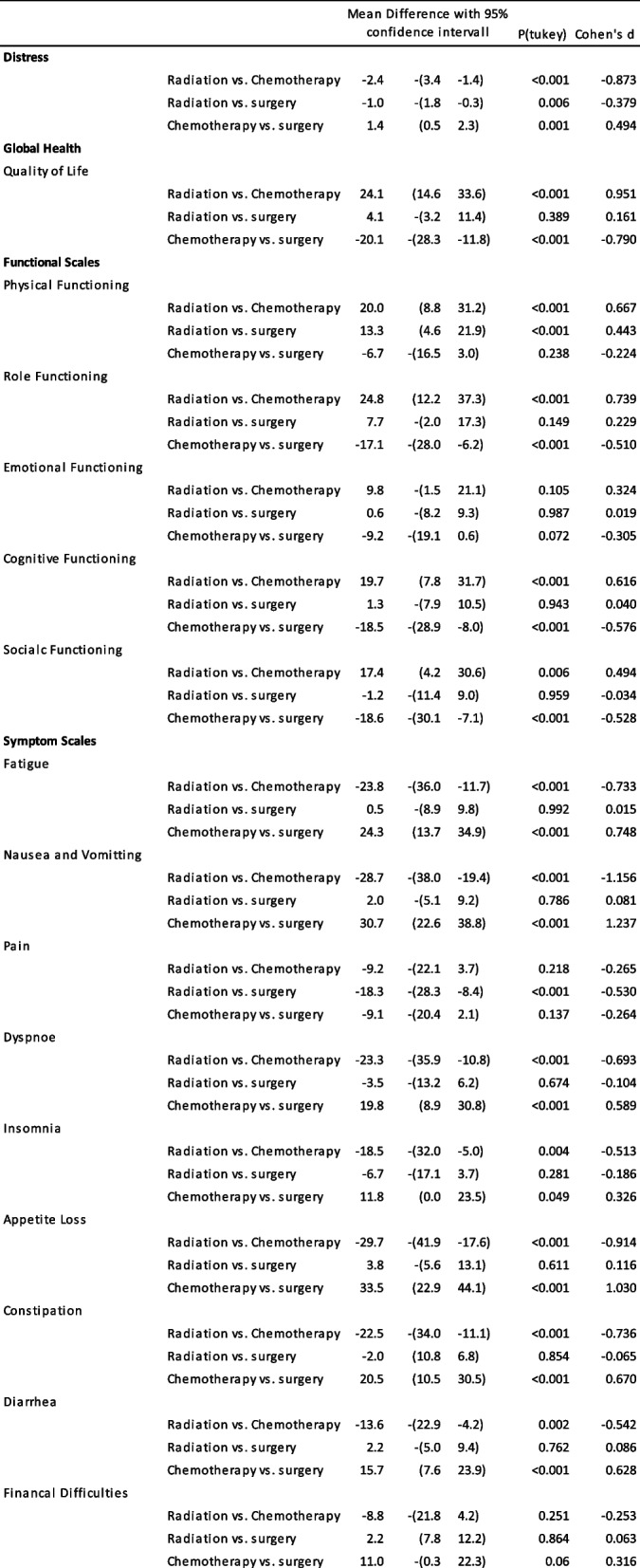
Mean difference between modality groups

#### EORTC—functional scales

Encompassing physical functioning, role functioning, emotional functioning, cognitive functioning, and social functioning. Physical functioning was significantly highest during radiotherapy, with a mean score of 63.5 [25.6] (F (2,176) = 13.490, *p* < 0.001, eta squared = 0.050). In contrast, both operation (mean = 50.3 [32.8]) and chemotherapy (mean = 43.5 [25.4]) exhibited significantly lower scores, although they were not significantly different from each other. This pattern was also consistent for the other functional scales as well (except emotional functioning), with chemotherapy having a more pronounced effect on well-being, resulting in significantly lower scores compared to operation and chemotherapy. However, there were no significant differences between surgery and chemotherapy. The *p* values for emotional functioning indicated no significant difference between the three modalities. Detailed values for role functioning, emotional functioning, cognitive functioning, and social functioning can be found in Tables 2 and 3.

#### EORTC—symptom scales

Notably, when it came to pain (F (2,385) = 9.703, *p* < 0.001, eta squared = 0.048), surgery (mean = 50.1 [36.0]) was significantly more discomforting than radiotherapy (mean = 31.8 [32.5]) and chemotherapy (mean = 40.9 [32.6]). However, radiation and chemotherapy did not exhibit a significant difference (Table 3).

Insomnia and sleep disturbances (F (2,164) = 5.813, *p* < 0.001, eta squared = 0.027) were reported predominantly during chemotherapy, with a mean score of 51.5 [36.8]. This score was significantly higher compared to radiation, which had a mean score of 33.0 [33.2]. There were no significant differences between surgery (mean = 39.7 [37.6) and chemotherapy in this regard.

Nausea and vomiting (F (2,385) = 41.473, *p* < 0.001, eta squared = 0.177) were primarily associated with chemotherapy (mean = 41.2.0 [36.5), while the values for radiation (mean = 12.5 [21.2]) and surgery (mean = 10.4 [21.8]) were significantly lower.

Regarding symptom scales related to fatigue, dyspnoea, appetite loss, constipation, and diarrhoea, a pattern similar to that seen in some of the functional scales mentioned earlier emerged. Chemotherapy resulted in significantly higher symptom scores compared to radiotherapy and operation, which did not exhibit significant differences between them.

Financial difficulties (F (2,151) = 2.346, p 0.099, eta squared = 0.013) were reported at a similar level across all three modalities, with no significant differences detected in the values of this scale.

## Discussion

A major limitation of this study is that the patients’ reports were not cross-checked or substantiated with clinical data obtained from the clinic systems. As a result, there is some ambiguity in interpreting factors such as chemotherapy and radiotherapy fractions. It is challenging to definitively discern whether the patients were referring to the frequency of visits or the actual number of fractions, as typically determined by healthcare experts. For example, three-fourths of all patients undergoing radiotherapy reported completing more than 15 fractions, with approximately 30% undergoing over 30 fractions. Due to anonymization, we were unable to verify this information.

Additionally, patients were responsible for selecting the correct sarcoma subtype diagnosis from a list. However, it’s important to note that a pre-selection process was carried out, and individuals who clearly did not meet the inclusion criteria were not initially included in the study.

Patients undergoing chemotherapy reported significantly higher levels of distress and a lower quality of life compared to the other two treatment modalities. This observation directly correlates with the percentage of patients who would choose the same treatment again, with chemotherapy receiving the lowest preference and surgery the highest.

It may initially appear surprising that the Retake Score for radiotherapy was lower than that for surgery, given some functional and symptomatic impairments associated with surgery. However, we believe that this finding is influenced by the psychological aspect of “eliminating the tumor.” Despite the potential pain and distress associated with surgery (as indicated in the results), patients perceive this treatment as the only genuine curative option and a chance to rid themselves of the malignancy.

A key emphasis was placed on examining the degree of patient impairment arising from therapy within the context of palliative care. In this study, patients rated chemotherapy significantly lower in terms of their quality of life compared to radiation therapy or surgery. Moreover, not only did quality of life suffer, but their functional well-being was also notably lower compared to the other two treatment modalities. A limitation of the study is that we did not exclusively survey patients in a palliative setting. In fact, it would have been most informative to question individuals who have passed away to obtain valid information about their preferences for treatment. However, due to practical constraints, we designed the study to approximate what was feasible within our available resources. This approach is particularly important given the relatively low incidence of these tumors, which results in small numbers of potential subgroups for analysis. Based on the data we gathered, given the relatively little impact on quality of life and low levels of distress, it could be inferred that while not curative, surgery may still provide relief for patients in a palliative care setting and should be considered.

Eichler et al. recently demonstrated a notable decrease in the quality of life, psychological well-being, and physical health of sarcoma patients. Their study also highlighted a heightened impairment among patients undergoing palliative care. Furthermore, it was observed that patients receiving surgery, radiotherapy, and chemotherapy experienced the most pronounced deficiencies. However, Eichler et al. did not differentiate between the well-being of patients undergoing the different therapies [18].

In this study, we differentiated between the various therapies. Although some patients underwent multiple treatments, they were counted separately for each therapy option. The EORTC data presented above were also analyzed for the group of patients who underwent all three main therapies. Unfortunately, the sample size of this group was insufficient to yield statistically significant results. Nevertheless, we observed the same trend in almost all measures as in the entire study population.

The observation of Eichler et. al. echoes findings across numerous studies on the quality of life in sarcoma patients [19]. Our data indicate that especially chemotherapy has the greatest negative impact on the quality of life of sarcoma patients. This finding might reflect, that chemotherapy is a systemic treatment, whereas radiation and surgery are localized therapies. In this study, the two primary chemotherapy drugs prescribed for STS were doxorubicin (63% of all patients receiving chemotherapy) and ifosfamide (54%). Gemcitabine (6%), Etoposid (3%), Cyclophosphamide (1%) and Paclitaxel (8%) were significantly less reported. Only 5% of all patients in this study treated with chemotherapy reported on a treatment with targeted drugs. Since specific drugs tailored to the various subtypes of sarcoma are lacking, doxorubicin and ifosfamide have remained the universally employed agents for nearly all soft tissue sarcoma subtypes for over a decade [20]. Recent therapeutic approaches that incorporate tyrosine inhibitors, as demonstrated in studies like Kummar S et al.’s work on cediranib for metastatic alveolar soft part sarcoma [21] and Stacchiotti S, Negri T, Zaffaroni N, et al.’s investigation into sunitinib [22] for advanced alveolar soft part sarcoma, hold promise. Escpecially in STS Pazopanib and Atezolizumab have shown quite good response rates [23]. Tazemetostat targets epitheloide sarcoma and TRK-Inhibitors like Larotrectinib and Entrectinib may be used for tumours inheriting NTRK-Gen-Fusion [24, 25].

However, these therapies have not yet been widely utilized for sarcoma treatment during our study and as a result, they were not a focus of our questionnaire.

Given the ongoing shift towards more personalized therapies, there is a need for further studies examining the quality of life during these novel treatments. In comparison to traditional chemotherapeutic agents, these new therapies may show other side effects and may be rated more favorably by patients in terms of their quality of life and physical well-being [21, 26, 27].

While surgery may not always result in a cure, even within a palliative framework, the removal of the sarcoma or significant portions of the tumor can provide relief for patients. This study demonstrates that the impact on quality of life from surgery is relatively less pronounced compared to chemotherapy. Moreover, the rates of repeat surgery (willingness to undergo the exact same procedure again) were notably higher in comparison to both chemotherapy and radiation, suggesting a significant positive influence on patients’ well-being. The data potentially contributes valuable evidence to the shared decision-making process with the patients and the tumour board discussions when determining therapy.

## Conclusion

In this research, we conducted interviews with 237 sarcoma patients to gain insight into their therapy experiences. We focused on the three most prevalent treatment modalities: chemotherapy, radiotherapy, and surgery. The results revealed that patients generally found chemotherapy to be the most challenging in terms of its impact on their quality of life, physical well-being and symptom management. Radiation therapy, on the other hand, was well-tolerated by most patients and despite the pain associated with surgery, nearly all patients would consent to surgery again because of limited side effects.

When evaluating these three therapeutic approaches, it’s important to note that chemotherapy typically involves ongoing treatment spanning months or even years. In contrast, radiation and surgery are generally completed within a few weeks. Additionally, chemotherapy works systemically, unlike radiation and surgery, which are more localized. Therefore, supportive therapies provided during both inpatient and outpatient care are particularly crucial. Nevertheless, given the significant negative effects of chemotherapy on patients’ quality of life, the extent to which classical chemotherapeutic agents are utilized in the palliative care setting must be carefully weighed in discussion with the respective patient.

## Data Availability

No datasets were generated or analysed during the current study.
